# Correlation of Body Mass Index and Waist-Hip Ratio with Severity and Complications of Hyperlipidemic Acute Pancreatitis in Chinese Patients

**DOI:** 10.1155/2017/6757805

**Published:** 2017-02-23

**Authors:** Lixin Yang, Jing Liu, Yun Xing, Lichuan Du, Jing Chen, Xin Liu, Jianyu Hao

**Affiliations:** ^1^Department of Gastroenterology, Beijing Chaoyang Hospital, Capital Medical University, Beijing, China; ^2^Department of Internal Medicine, Beijing First Hospital of Integrated Chinese and Western Medicine, Beijing, China; ^3^Department of Internal Medicine, Hebei Xianghe People's Hospital, Hebei Province, China

## Abstract

Hyperlipidemic acute pancreatitis (HLAP) is characterized by critical condition and high recurrence rate compared with non-HLAP. We conducted this study to investigate the value of body mass index and waist-hip ratio in predicting severity and local complications in HLAP. 96 patients with HLAP were categorized by body mass index and waist-hip ratio, respectively. According to the body mass index, they were divided into 3 groups, including normal weight, overweight, and obesity. According to the waist-hip ratio, they were divided into central obesity group and no central obesity group. The body mass index and waist-hip ratio were compared in severity, local complications, and systematic complications of HLAP, using chi-square test and Monte Carlo simulations. The body mass index and waist-hip ratio were correlated with the severity of acute pancreatitis (MAP, MSAP, and SAP), respiratory failure, and circulatory failure in HLAP (*p* < 0.05), but not correlated with the local complications (walled-off necrosis, pancreatic abscess, and pancreatic pseudocyst), renal failure, and gastrointestinal bleeding.The body mass index and waist-hip ratio are valuable in predicting severity and complication in HLAP. We demonstrated that obese patients had an increased risk of developing more serious condition and more complications in HLAP.

## 1. Introduction

Recently, with the change of people's living condition and lifestyle, hyperlipidemia comes to the second most common cause of acute pancreatitis. A study involving 2416 diagnosed acute pancreatitis (AP) cases from 2006 to 2010 in Beijing found that 255 (10.36%) cases were hyperlipidemic acute pancreatitis (HLAP) [1]. An analysis carried out by Xu et al. claims that in the period of 2012 to 2014, HLAP accounted for 19.1% of total AP [2]. Compared with NHLAP, HLAP are characterized by critical condition and high recurrence rate [3].

Several studies have shown that obesity is an indicator for severity of acute pancreatitis [4, 5]. However, to the best of our knowledge, few studies exist investigating the relationship between obesity and HLAP using the definition of obesity for Chinese population. The aim of this study is to explore the correlation between body mass index and waist-hip ratio and the classification of severity and the incidence of local complications and systemic complications in HLAP. And we investigate the assessment value of body mass index and waist-hip ratio in predicting severity of HLAP at an early stage.

## 2. Materials and Methods

We retrospectively analyzed a series of 96 patients diagnosed with HLAP who were admitted to Beijing Chaoyang Hospital, Capital Medical University, in a period of June 2011 to January 2014 (53 males, 43 females, age ranges from 17 to 76, mean age 41 years). Of the 96 patients included, 38 were normal weight, 27 were overweight, and 31 were obesity; 55 were noncentral obesity; and 41 were central obesity. Among them, 72 had mild acute pancreatitis (MAP), 16 had moderately severe acute pancreatitis (MSAP), and 8 had severe acute pancreatitis (SAP), of which 3 patients with SAP died (3.1%).

Body mass index (BMI) is a value derived from the weight and height of an individual. The BMI is defined as the body mass divided by the square of the body height and is universally expressed in units of kg/m^2^, resulting from mass in kilograms and height in meters. According to the criteria in the guidelines for prevention and control of overweight and obesity in Chinese adults, normal weight, overweight, and obesity were defined as BMI < 24 kg/m^2^, 24 ≤ BMI < 28 kg/m^2^, and BMI ≥ 28 kg/m^2^, respectively.

Waist to hip ratio is the ratio of the circumference of the waist to that of the hips. It is an important measure of central obesity. Waist circumference is measured at a level midway between the lowest rib and the iliac crest. The tape measure was in a plane parallel to the floor and was snugged without compressing the skin. The hip circumference was also measured using the same measuring tape at its widest portion of the buttocks, with the tape parallel to the floor. Waist circumference and hip circumference were measured to the nearest 0.1 cm. All of the patients were divided into the central obesity group (male > 0.90, female > 0.85) and no central obesity group (male < 0.90, female < 0.85) [6, 7].

The diagnostic criteria of acute pancreatitis referred to the Chinese guideline for diagnosis and treatment of acute pancreatitis [8]. HLAP was diagnosed if serum triglycerides (TG) reached 11.3 mmol/L or TG was more than 5.56 to 11.3 mmol/L accompanied by chylemia, excluding other etiologies of AP (gallstone, drug, infection, etc.) [9, 10].

The data was statistically analyzed using SPSS software 19.0. The BMI and WHR were compared in severity, local complications (walled-off necrosis, pancreatic abscess, and pancreatic pseudocyst), and systematic complications (respiratory failure, circulatory failure, renal failure, and gastrointestinal bleeding) of HLAP, using the chi-square test and Monte Carlo simulations. A *p*  value < 0.05 was considered statistically significant.

We obtained approval from the Institutional Review Board of Beijing Chaoyang Hospital for this study.

## 3. Result

### 3.1. Comparisons of the Severity of HLAP in Different Groups

No significant statistical difference was found related to sex and age with the severity and complications of HLAP between the normal weight group, overweight group, and obesity group, as well as the central obesity group and no central obesity group (*p* > 0.05).

As shown in Table 1, among 96 patients included, 8 cases developed SAP (1 of normal weight, 2 of overweight, and 5 of obesity) and 16 cases developed MSAP (3 of normal weight, 4 of overweight, and 9 of obesity). There were significant differences among the three groups (*p* < 0.05).

The comparison of the no central obesity group (*n* = 55) versus the central obesity group (*n* = 41) was as follows: MAP, 47 versus 25; MSAP, 6 versus 10; and SAP, 2 versus 6. There were significant differences between the two groups (*p* < 0.05).

### 3.2. Comparisons of the Complications of HLAP in Different Groups

There were no significant differences in the incidence of local complications (walled-off necrosis, pancreatic abscess, and pancreatic pseudocyst), renal failure, and gastrointestinal bleeding between the normal weight group, overweight group, and obesity group (*p* > 0.05), as well as between the no central obesity group and central obesity group (*p* > 0.05).

But the incidence of respiratory failure and circulatory failure was significantly different between the normal weight group, overweight group, and obesity group (*p* < 0.05). The patients with central obesity had a significant higher incidence of respiratory failure and circulatory failure when compared with patients without central obesity (*p* < 0.05) (Table 2).

There were significant differences in the incidence of hypertension and diabetes mellitus between the normal weight group, overweight group, and obesity group (*p* < 0.05), as well as between the no central obesity group and central obesity group (*p* < 0.05). The patients with higher BMI or central obesity had a significant higher incidence of hypertension and diabetes mellitus (Table 3).

There was an insignificant relationship between serum triglycerides and the incidence of local complications (walled-off necrosis, pancreatic abscess, and pancreatic pseudocyst), respiratory failure, renal failure, and circulatory failure (*p* < 0.05). Patients with serum TG more than 11.3 mmol/L had a higher incidence of local complications, respiratory failure, renal failure, and circulatory failure than those with TG more than 5.56 to 11.3 mmol/L.

## 4. Discussion

It has been reported that the incidence of SAP, organ dysfunction, recurrence rate, and mortality of HLAP was significantly higher than that of acute biliary pancreatitis [3]. Hyperlipidemia may induce acute pancreatitis through mechanisms of inflammatory response, microcirculation disorder, oxidative stress, calcium overload, metabolic abnormalities, and so forth. Compared with NHLAP, HLAP has the following features: (1) high recurrence rate—the higher the blood lipid level is, the greater the possibility of recurrence. Serum TG lower than 5.56 mmol/L may prevent development of pancreatitis. (2) Serum TG is above 11.3 mmol/L. (3) Because of lipid deposition, xanthomata in the limbs, buttocks, and back; retinal lipemia; hepatosplenomegaly; and fatty liver can be found in patients with severe hypertriglyceridemia (HTG). (4) Patients with HLAP have younger age of onset. Uncontrolled diabetes, obesity, alcoholism, pregnancy, and family history of hyperlipidemia are thought to be the risk factors for HLAP [11, 12]. So, it is crucial to identify the risk factors for reducing the severity and complications of HLAP.

Metabolic syndrome, characterized by multiple organ damage, is a clustering of central obesity, lipid metabolism disorders, type 2 diabetes, and hypertension. With the increasing incidence of metabolic syndrome, it becomes more and more common that acute pancreatitis coexist with metabolism syndrome. In 1990, BMI was first introduced as a prognostic predictor of acute pancreatitis by Lankisch and Schirren. They demonstrated that because of metabolic disorder, obese patients had an increased incidence of chronic diseases, such as cholecystitis, gallstone disease, hypertension, and hyperlipidemia. Furthermore, obesity is correlated with hyperlipidemia. And some of them contributed to the episode and exacerbation of acute pancreatitis [13]. It has been shown that metabolism syndrome was correlated with the severity of AP [14].

Obesity is a predictor of occurrence and severity form of acute pancreatitis. A study by De Waele et al. showed that overweight and class I, II, and III obese patients are at increased risk of developing the severe form of disease when compared with normal-weight patients [15]. Patients with central obesity have lots of fat accumulated around abdominal organs. The fat deposits around the pancreas provide the material for pancreatic necrosis and saponification. A large amount of free fatty acids generated from fat lipolysis directly damage the gland cells and capillaries of the pancreas. Pancreatic capillary injury leads to local ischemia and acidic environment, which enhance the toxicity of free fatty acids. Adipose tissue is an endocrine organ that secretes a variety of inflammatory cytokines. In obese patients, fat necrosis and hypoxia release inflammatory factors that accelerate SIRS and multiple organ failure. Obesity makes an increase in severity of AP through a mechanism of IL6, IL8, and other inflammatory mediators. Obese patients with acute pancreatitis had more critical condition, as well as a higher incidence of local and systemic complications [16, 17].

In this study, BMI and WHR were used as the measures of obesity. According to the definition of obesity for Chinese population, we confirmed that higher BMI and WHR were correlated with the increased risk of more serious form of acute pancreatitis, respiratory failure, and circulatory failure in patients who were diagnosed with HLAP. But BMI and WHR were not correlated with the local complications, renal failure, and gastrointestinal bleeding. Respiratory failure and circulatory failure might be linked to a large secretion of mediators of inflammation from peripancreatic fat necrosis. These cytokines contribute to the systemic inflammatory response syndrome and lead to respiratory failure and circulatory failure.

We also found that patients with HLAP who had higher BMI and central obesity had an increased incidence of hypertension and diabetes. History of hypertension was correlated with respiratory failure and circulatory failure. History of diabetes was correlated with respiratory failure. And there was a strong relationship between the levels of serum triglycerides and local complications and systematic complications in HLAP. The higher the serum triglycerides, the higher the risk of local complications and systematic complications.

However, no statistically significant increase in risk of local complications was observed in this study. Due to the small sample size and the low incidence of local complications, we thought complementary studies focused on this aspect should be done. In addition, further studies should investigate whether central obesity, as measured by WHR, predicts risk independent of overall obesity, as measured by BMI.

In conclusion, our results support an association between obesity and the severity of HLAP. We suggested that body mass index and waist-hip ratio may predict the severity and complications in HLAP. Therefore, we suggested combining traditional scoring systems (BISAP, Ranson, APACHE II, CTSI, etc.) with BMI and WHR might improve the diagnostic accuracy. And that is the direction of our further studies.

## Figures and Tables

**Table 1:** Correlation of BMI and WHR with severity in HLAP.

		MAP (*n* = 72)	MSAP (*n* = 16)	SAP (*n* = 8)	Total	*p* value
BMI	Normal	34	3	1	38	*p* < 0.05
	Overweight	21	4	2	27	
	Obesity	17	9	5	31	

WHR	No central obesity	47	6	2	55	*p* < 0.05
	Central obesity	25	10	6	41	

**Table 2:** Correlation of BMI and WHR with complications in HLAP.

		BMI				WHR		
		Normal	Overweight	Obesity	*p* value	No central obesity	Central obesity	*p* value
Local complications	Walled-off necrosis (*n* = 5)	1	1	3	NS	2	3	NS
	Abscess (*n* = 5)	1	2	2	NS	2	3	NS
	Pseudocyst (*n* = 8)	2	3	3	NS	3	5	NS

Systemic complications	Respiratory failure (*n* = 14)	2	3	9	*p* < 0.05	4	10	*p* < 0.05
	Renal failure (*n* = 6)	1	2	3	NS	2	4	NS
	Circulatory failure (*n* = 16)	2	4	10	*p* < 0.05	4	12	*p* < 0.05
	Gastrointestinal bleeding (*n* = 8)	2	3	3	NS	3	5	NS

NS = not significant.

**Table 3:** Correlation of history of hypertension and diabetes and serum triglycerides with complications in HLAP.

		History of hypertension			History of diabetes			Serum triglycerides		
		No	Yes	*p* value	No	Yes	*p* value	5.65–11.3 mmol/L	>11.3 mmol/L	*p* value
Local complications	Walled-off necrosis (*n* = 5)	2	3	NS	3	2	NS	0	5	*p* < 0.05
	Abscess (*n* = 5)	3	2	NS	1	4	NS	1	4	*p* < 0.05
	Pseudocyst (*n* = 8)	4	4	NS	3	5	NS	1	4	*p* < 0.05

Systemic complications	Respiratory failure (*n* = 14)	5	9	*p* < 0.05	4	10	*p* < 0.05	2	12	*p* < 0.05
	Renal failure (*n* = 6)	3	3	NS	2	4	NS	1	5	*p* < 0.05
	Circulatory failure (*n* = 16)	5	11	*p* < 0.05	7	9	NS	3	13	*p* < 0.05
	Gastrointestinal bleeding (*n* = 8)	4	4	NS	3	5	NS	3	5	NS

NS = not significant.
